# Daily variation of NTBC and its relation to succinylacetone in tyrosinemia type 1 patients comparing a single dose to two doses a day

**DOI:** 10.1007/s10545-017-0112-9

**Published:** 2017-11-23

**Authors:** Nienke S. Kienstra, Hannah E. van Reemst, Willem G. van Ginkel, Anne Daly, Esther van Dam, Anita MacDonald, Johannes G. M. Burgerhof, Pim de Blaauw, Patrick J. McKiernan, M. Rebecca Heiner-Fokkema, Francjan J. van Spronsen

**Affiliations:** 1 Department of Metabolic Diseases, Beatrix Children’s Hospital, University Medical Center Groningen, University of Groningen, Hanzeplein 1, 9700 RB Groningen, The Netherlands; 2 Department of Metabolic Diseases, Birmingham Children’s Hospital, Birmingham, UK; 3 Department of Dietetics, University Medical Center Groningen, University of Groningen, Groningen, The Netherlands; 4 Department of Epidemiology, University Medical Center Groningen, University of Groningen, Groningen, The Netherlands; 5 Department of Laboratory Medicine, University Medical Center Groningen, University of Groningen, Groningen, The Netherlands

**Keywords:** Tyrosinemia type 1, NTBC, Succinylacetone, SA

## Abstract

**Introduction:**

In hereditary tyrosinemia type 1 (HT1) patients, the dose of NTBC that leads to the absence of toxic metabolites such as succinylacetone (SA) is still unknown. Therefore, the aims of this study were to investigate the variation and concentrations of 2-(2-nitro-4-trifluormethyl-benzyl)-1,3-cyclohexanedione (NTBC) during the day in relation to the detection of SA, while comparing different dosing regimens.

**Methods:**

All patients were treated with NTBC (mean 1.08 ± 0.34 mg/kg/day) and a low phenylalanine-tyrosine diet. Thirteen patients received a single dose of NTBC and five patients twice daily. Home bloodspots were collected four times daily for three consecutive days measuring NTBC and SA concentrations. Statistical analyses were performed by using mixed model analyses and generalized linear mixed model analyses to study variation and differences in NTBC concentrations and the correlation with SA, respectively.

**Results:**

NTBC concentrations varied significantly during the day especially if NTBC was taken at breakfast only (*p* = 0.026), although no significant difference in NTBC concentrations between different dosing regimens could be found (*p* = 0.289). Momentary NTBC concentrations were negatively correlated with SA (*p* < 0.001). Quantitatively detectable SA was only found in subjects with once daily administration of NTBC and associated with momentary NTBC concentrations <44.3 μmol/l.

**Discussion:**

NTBC could be less stable than previously considered, thus dosing NTBC once daily and lower concentrations may be less adequate. Further research including more data is necessary to establish the optimal dosing of NTBC.

**Electronic supplementary material:**

The online version of this article (10.1007/s10545-017-0112-9) contains supplementary material, which is available to authorized users.

## Introduction

Hereditary tyrosinemia type 1 (HT1; McKusick 276,700) is a rare, autosomal recessive metabolic disorder caused by a deficiency of fumarylacetoacetate hydrolase, the last enzyme in the tyrosine catabolism pathway. This deficiency results in accumulation of toxic metabolites, such as maleylacetoacetate, fumarylacetoacetate (FAA), succinylacetoacetate, and succinylacetone (SA). The accumulation of these toxic metabolites can cause liver failure, hepatocellular carcinoma (HCC), renal tubulopathy, cardiomyopathy, and porphyria-like-syndrome with neuropathy (van Spronsen et al 1994, Larochelle et al 2012, de Laet et al 2013). Life expectancy in HT1 patients is low when they are treated with a low phenylalanine and tyrosine diet only (van Spronsen et al 1994, de Laet et al 2013).

The course of the disease has changed remarkably since 1992, when it was observed that 2-(2- nitro-4-trifluormethyl-benzyl)-1,3-cyclohexanedione (NTBC) prevents the accumulation of toxic metabolites by blocking the tyrosine degradation at the level of 4-OH-phenylpyruvate dioxygenase, proximal from the primary enzymatic defect (Lindstedt et al 1992). This in turn prevents the formation of toxic products, leading to resolution of liver failure and porphyria-like-syndrome and a substantial reduction in HCC (Holme and Lindstedt 1998, Holme and Lindstedt 2000). Consequently, life expectancy in HT1 has considerably improved (Larochelle et al 2012). However, as NTBC blocks the tyrosine degradation pathway, tyrosine concentrations increase, so restriction of dietary tyrosine and phenylalanine is still necessary (Russo et al 2001, de Laet et al 2013).

Despite improved treatment with NTBC, previous reports indicate that HT1 patients are still at risk for developing hepatocellular carcinoma (van Spronsen et al 2005, Koelink et al 2006, van Ginkel et al 2015). This may be related to suboptimal treatment with NTBC. Accumulation of FAA causes the development of HCC (Jorquera and Tanguay 1997, Jorquera and Tanguay 1999). As SA can be used as a surrogate marker of toxicity in the liver, we should aim at the lowest NTBC dose associated with the lowest possible SA concentration in blood and urine (Grompe et al Grompe 2001, de Laet et al 2013, Mayorandan et al 2014).

The current dosing recommendation is twice a day as specified by the manufacturers (Orfadin [package insert] 2017). However, a single dose of NTBC per day was suggested in HT1 patients (at 1 mg/kg/day), based on the long half-life of 54 h observed in healthy adults and the finding that in HT1 patients NTBC concentrations tended to be stable for at least 24 h with a single dose regime (Hall et al 2001, Schlune et al 2012, de Laet et al 2013).

However, in practice, blood NTBC levels are reported to vary greatly between individuals (Schlune et al 2012, de Laet et al 2013) and within the same patients with time. As a consequence, target blood NTBC concentrations are not well established. Therefore, the research questions of this study were: (1) Do mean NTBC concentrations and daytime variation of NTBC differ between different NTBC dosing regimens? (2) What is the occurrence of elevated SA with different NTBC dosing regimens? Answering these research questions may help to suggest an optimal dosing regimen and to indicate a minimal NTBC concentration associated with the absence of toxic metabolites represented by SA.

## Methods

### Subjects

In total, 18 HT1 patients (13 males, five females; mean age 9.3 ± 6.8 years; range 1–20 years) were studied. Five patients were diagnosed and treated in the University Medical Center Groningen and 13 patients in the Birmingham’s Children’s hospital (UK). Except for having HT1, patients were healthy, did not have signs suggestive of liver dysfunction, HCC or renal tubulopathy. Patients with HT1 were included when they were older than one year of age and treated with NTBC and a tyrosine and phenylalanine restricted diet. All subjects maintained their regular NTBC dose and dosing regime and diet with individually tailored natural protein intake, titrated according to target blood tyrosine concentrations (200–400 μmol/L) during the study period. In five patients, the total daily dose of NTBC was divided into two doses, while the other 13 patients were given a single daily dose of NTBC. Stable dietary protein and energy intakes were maintained. Phenylalanine supplementation was not prescribed to any patient during the study period.

The study was approved by the medical ethical committee of the University Medical Center Groningen in The Netherlands and a favorable opinion was given by the South Birmingham ethical committee for Birmingham Children’s Hospital, UK. All HT1 caregivers gave written informed consent for this study and children gave assent if age and understanding was appropriate.

### Study design

In this observational prospective study, patients or the caregivers of patients took blood spot samples on blood cards at home four times daily (pre-breakfast, pre-midday meal, pre-evening meal, and pre-bedtime) for three consecutive days. In total, 12 blood spots were taken for each subject and overall, 214 samples were collected (from two subjects, one sample was not obtained). The caregivers or patients were trained to take blood spots themselves.

### Analyses of blood results

All blood spots were stored in a sealed plastic bag with a silica sachet at −20 °C until analysis. Blood spot NTBC and SA concentrations were measured in the University Medical Center Groningen using an ultra-high performance liquid chromatography method coupled to a triple quadrupole mass spectrometer (UHPLC-MS/MS). The detection limit of blood spot SA was 0.2 μmol/l with a limit of quantification of 0.6 μmol/L. A detailed description of the method, including validation, is provided as Supplementary material.

### Statistics

Baseline differences between the different treatment groups (NTBC taken at breakfast, evening meal or both) were studied using one-way ANOVA analysis. The variation of NTBC during the day was studied using mixed model analyses, by considering the moment of NTBC intake (at breakfast, evening meal or both), the moment of blood sampling and an interaction between both. To further study differences in NTBC concentrations during the day between subjects with different treatment regimens (taking NTBC at breakfast, at evening meal or both) univariate mixed model analysis was done. To study the correlation between momentary NTBC and SA concentrations ≥0.6 μmol/L, generalized linear mixed model analyses was performed. Afterwards, mean NTBC concentrations during the study period and the number of samples with SA ≥ 0.6 μmol/L per patient were calculated and Spearman correlational analyses were performed between both variables. In addition, differences in mean NTBC concentrations for patients with and without quantitatively detectable SA during the study period were analyzed using an independent sample T-test. Statistical analyses were conducted with the statistical program SPSS 22 (IBM, Chicago, Illinois). A *p*-value of <0.05 was considered statistically significant.

## Results

**Fig. 1:**
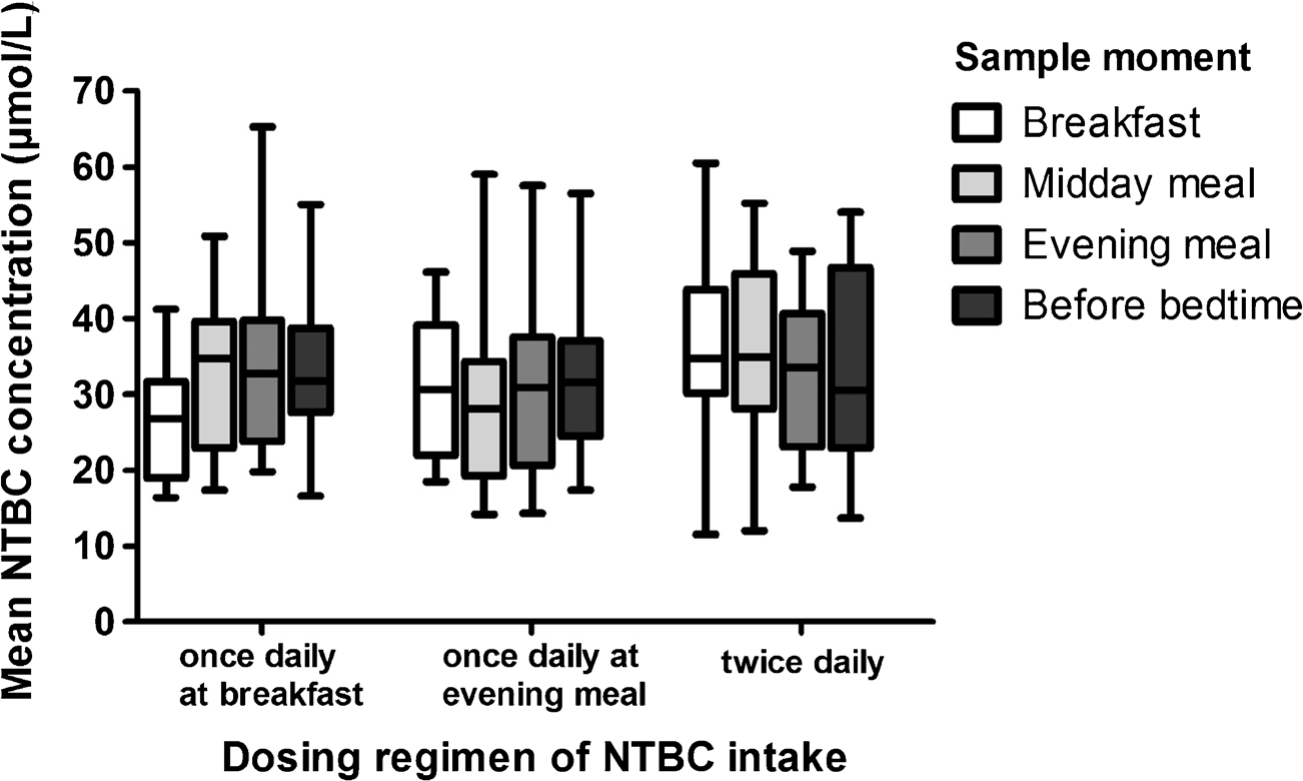
Mean NTBC concentrations at the different sample moments during the day. Mean NTBC concentrations during the day in the group of HT1 patients, divided based on different treatment regimes. Especially showing variation in the group of patients taking their NTBC at breakfast only

Subject characteristics are given in Table 1. The subjects were divided into three groups depending on their moment of intake of NTBC. For the group who received NTBC once daily (*N* = 13), six subjects received NTBC at breakfast and seven subjects received NTBC at the evening meal. The subjects taking NTBC twice daily received their NTBC both at breakfast and at the evening meal. The total daily NTBC dose (in mg/kg/day) did not significantly differ between the three groups of subjects (subjects receiving NTBC in a single dose or in two doses) (*p* = 0.13). Despite that natural protein intake, total protein intake, and tyrosine concentrations seemed to differ, none reached statistical significance (*p* = 0.12, p = 0.13, and p = 0.13, respectively). Figure 1 shows the variation of NTBC during the day, comparing subjects who received a single dose of NTBC, at breakfast or evening meal, and subjects who divided their NTBC in two doses a day. Mixed model analyses showed a significant interaction between the timing of NTBC intake and the moment of blood sampling (*p* = 0.041), indicating that the pattern of NTBC concentrations during the day was different between the three different groups. Post hoc analyses showed that the group of subjects taking NTBC at breakfast had a significant variation of NTBC concentrations especially in the morning (*p* = 0.026). No significant variation in NTBC concentrations during the day was seen in both other groups (subjects who took two doses of NTBC and subjects who took a single dose of NTBC at the evening meal).

**Table 1:** Patient characteristics for the different groups receiving NTBC as a single dose or divided in two doses a day

	HT1 NTBC once daily (*n* = 13)		HT1 NTBC twice daily (*n* = 5)
	NTBC at breakfast (*n* = 6)	NTBC at evening meal (*n* = 7)	
Mean age	8.5 (± 4.1 year)	8.8 (± 3.9 year)	9.9 (± 7.4 year)
Gender	4:2 (m/f)	4:3 (m/f)	4:1 (m/f)
NTBC intake (mg/kg/day)	1.14 ± 0.50	0.99 ± 0.30	1.15 ± 0.24
Total protein intake (g/kg/day)	2.59 ± 0.83	2.49 ± 0.55	1.72 ± 0.76
Natural protein intake (g/kg/day)	0.79 ± 0.32	0.73 ± 0.36	0.43 ± 0.06
Mean tyrosine (μmol/L)	356 ± 47.5	423 ± 98.6	312 ± 115
Mean phenylalanine (μmol/L)	40.5 ± 9.4	43.0 ± 10.5	39.0 ± 11.3

Figure 1 also shows the mean NTBC concentrations during the day comparing the three different groups of subjects. Mixed model analyses were done to compare NTBC concentrations of the three different subject groups at all four different timing moments of blood sampling during the day, but no significant differences were found. However, NTBC concentrations tended to be higher during the day when subjects received NTBC twice daily as also shown in Table 2. Of note, one patient lived a far distance from the clinical center. The blood spots were taken in a warm climate and transport took more time. This subject had very low levels of NTBC (mean = 16.8 ± 3.9) that deviated from the NTBC concentrations of other subjects who were treated in the same center and treated similarly (Table 2). When excluding this patient, the tendency toward differences in NTBC concentrations in the morning between the group taking NTBC at breakfast and the group taking NTBC twice daily became statistically significant (*p* = 0.016).

**Table 2:** Mean NTBC concentrations (+ SD) in μmol/L during the day in the different treatment groups, with significantly lower NTBC concentrations in the morning in the group of patient taking NTBC at breakfast only, compared to patients who take NTBC twice a day (after exclusion of outlier). *p < 0.05

	NTBC single dose treatment		NTBC two doses a day	
	NTBC at breakfast (n = 6)	NTBC at evening meal (n = 7)	All patients (n = 5)	Excluding outlier (n = 4)
Pre-breakfast sample	26.2 ± 7.8*	31.0 ± 8.9	33.8 ± 13.2	38.9 ± 8.9
Pre-midday sample	33.0 ± 9.7	30.5 ± 12.5	35.3 ± 12.0	39.9 ± 8.3
Pre-evening meal sample	34.4 ± 12.5	30.6 ± 11.4	32.5 ± 9.7	36.0 ± 7.3
Bedtime sample	33.6 ± 9.2	32.0 ± 10.1	33.8 ± 12.9	38.0 ± 10.8
Overall	31.8 ± 10.2	31.0 ± 10.6	33.8 ± 11.8	38.1 ± 8.7

No quantitatively detectable SA was found in subjects administered twice daily NTBC. However, SA ≥ 0.6 μmol/L was found in > 50% of subjects (seven of the 13 subjects) who took NTBC once daily. Three of them took their NTBC at breakfast and four subjects took their NTBC at the evening meal. In total, SA ≥ 0.6 μmol/L was found in 54 of 156 samples. The median SA concentration in the samples with SA above the limit of quantification was 0.7 μmol/L (range: 0.6–1.2 μmol/L). There was no significant difference in the age of subjects with SA concentrations ≥0.6 μmol/L and subjects who did not (*p* = 0.724). Neither was there a specific time during the day that SA could be found, as the number of samples with SA was equally distributed across the different sample moments during the day.

Generalized linear mixed model analyses showed that in individual samples, momentary NTBC concentrations were negatively correlated to SA concentrations ≥0.6 μmol/L(β = −0.062 *p* = 0.000). In the samples where SA was observed, the NTBC concentrations varied between 14.2–44.3 μmol/L. When the NTBC concentration in momentary samples were >44.3 μmol/L, no quantitatively detectable SA was found.

**Fig. 2:**
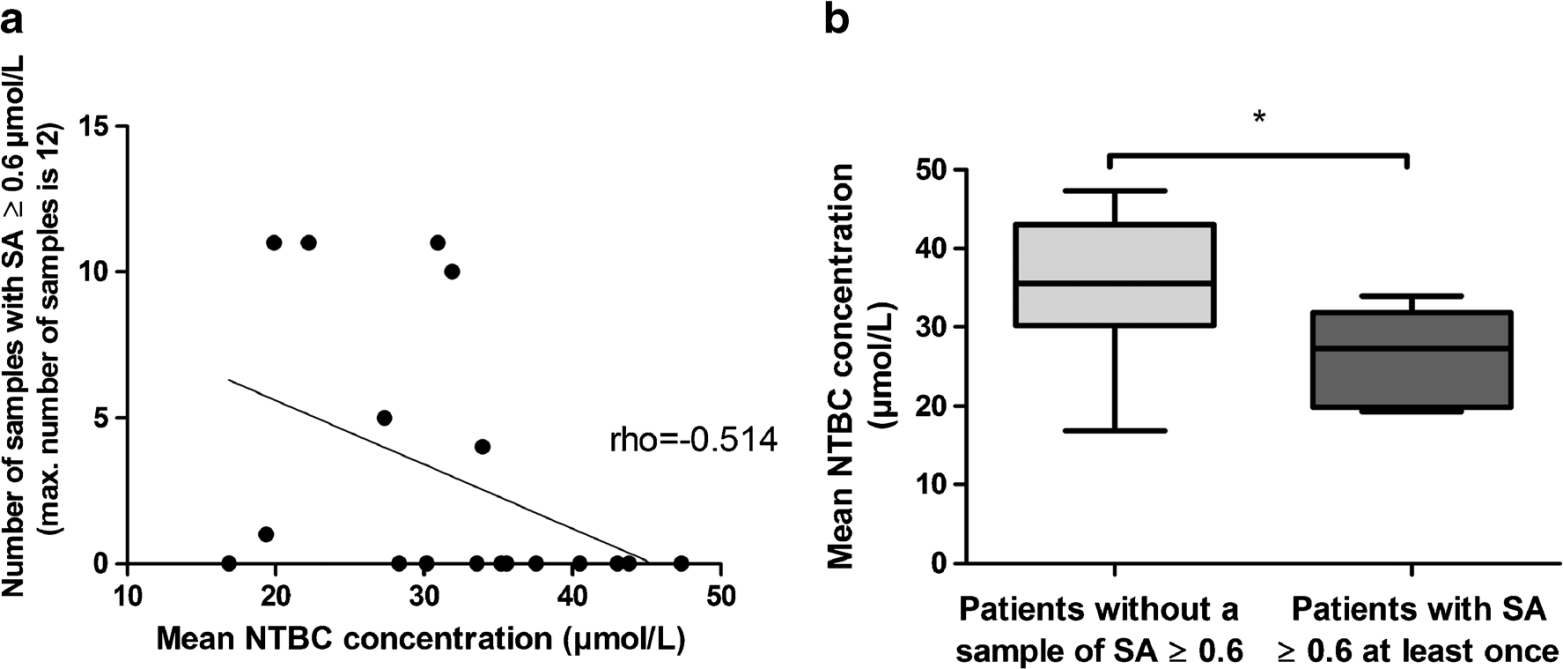
Mean NTBC concentrations and its relation to SA. **a **Mean NTBC concentrations during the study period graphically displaced against the number of samples with SA ≥ 0.6 μmol/L. The linear line represents the negative correlation between the number of samples with SA ≥ 0.6 μmol/L and mean NTBC concentrations. **b** Mean NTBC concentrations in patients with and without any sample with quantitatively detectable SA (≥ 0.6 μmol/L) during the study period

To further analyze the correlation between NTBC concentrations and the samples with quantitatively detectable SA, mean NTBC concentrations during the study period were calculated and related to the samples with quantitatively detectable SA. Figure 2a shows that if one sample in an individual patient showed a SA concentration above the detection limit of quantification, SA concentrations were usually quantifiable during a large part of the study. Correlational analyses revealed a significant negative correlation between the mean NTBC concentration during the study period and the number of samples with quantitatively detectable SA per patient (*ρ* = −0.514, *p* = 0.029). In addition, Fig. 2a shows that when mean NTBC concentrations during the study period were >35 μmol/L, no SA ≥ 0.6 μmol/L was found. Figure 2b shows that the mean NTBC concentrations during the study period were significantly higher in the subjects who did not show any quantitatively raised SA concentrations (mean = 35.6 ± 8.5) compared to subjects where SA was quantifiable at least once (mean = 26.5 ± 6.0) (*p* = 0.026).

## Discussion

With the dosing regimen of NTBC as advised by de Laet (de Laet et al 2013), increased SA concentrations are still found. This could possibly indicate a sub-optimal block, of course acknowledging the fact that not detecting SA may be due to technical reasons rather than the absence of SA. Therefore, the aims of this study were to investigate NTBC concentrations and its variation during the day and to study SA in relation to the NTBC dosage. By doing this, we investigated if a specific NTBC dosing regimen could be advised in HT1 patients. The main findings were: (1) NTBC concentrations varied during the day, particularly when NTBC was given once daily, and especially when taken at breakfast only. (2) NTBC concentrations tended to be higher and more stable in the patients receiving two doses of NTBC a day, but this did not reach statistical significance, and (3) NTBC concentrations were negatively correlated to SA. Quantitatively detectable SA was only found in patients who took one single dose of NTBC, especially when momentary NTBC concentrations were <44.3 μmol/L.

Before discussing the results in more detail, four methodological issues need to be addressed. Firstly, this study was an observational study and would have had a higher power if both treatment regimens were being tested in the same patients using a cross-over design. Secondly, the study was performed at patient’s homes. All blood spots were sent together after finishing the complete study and were analyzed at the University Medical Center Groningen and stored at −20 °C until analyzed. However, one of the patients receiving two daily doses of NTBC lived in a warm climate 3500 km from the center where he was treated, resulting in a time delay for receipt of blood spot samples. Although it was found that NTBC concentrations in blood spots were stable for at least a period of 1 month when stored at room temperature (La Marca et al 2012), our laboratory experience revealed that NTBC and SA concentrations are more stable when stored at low temperature. Further studies are needed to explore whether different storage conditions (like warm climate or a longer duration until analysis) influence NTBC and SA concentrations in dried blood spots. Thirdly, especially our findings on SA concentrations may be influenced by the limit of quantification rather than the exact SA concentration. Since most subjects had SA levels below limit of quantification using a lower limit of quantification for SA would have resulted in different findings. Fourth, although not statistically significant, total and natural protein intake in the group of patients taking NTBC once daily seemed to be higher compared to the group taking NTBC twice daily. Theoretically, higher protein intake could result in higher tyrosine concentrations and a higher flux through the tyrosine degradation pathway (Holme and Lindstedt 1998). However, tyrosine concentrations did not differ significantly as well.

Our results show a significant variation in NTBC concentrations during a 24-h period especially seen in patients who took their NTBC as a single dose at breakfast. In this group a drop of 21.3% in NTBC concentrations in < 20 h was observed between the pre-midday and pre-breakfast sample. This contrasts with previous studies that indicated NTBC had a stability of at least 24 h in HT1 patients, while in healthy adults even a half-life of 54 h was found (Schlune et al 2012, Hall et al 2001). The variation in NTBC concentrations in our sample at least suggests that biological availability was < 24 h and that this could have implications regarding the detection of SA, especially for subjects taking a single daily dose of NTBC.

When considering NTBC concentrations, our data indicate a tendency toward lower NTBC concentrations for the patients receiving NTBC once daily in comparison with receiving NTBC twice daily rather than a statistically significant difference. However, clear statistical significance was reached when the subject with the long transport time of samples was excluded as an apparent outlier probably due to differences in pre-analytical conditions. This is in contrast to previous studies, which failed to show any differences between dosing once, twice, and three times a day (Schlune et al 2012).

Both, momentary and mean NTBC concentrations during the study period were negatively correlated to SA. The difference between momentary and mean NTBC concentrations without quantitatively detectable SA is expected to be caused by the long half-life of SA in plasma (Holme and Lindstedt 1998). Quantitatively detectable SA possibly indicates a period of lower NTBC concentrations before the moment of blood sampling. Most importantly, in our study, SA was not found when momentary NTBC concentrations were above 44.3 μmol/L, which is in accordance with the results of Herebian et al (2009) who concluded that NTBC concentrations should be above 50 μmol/L (Herebian et al 2009).

Acknowledging the importance of maintaining SA concentrations as low as possible, our data seem to indicate that dosing NTBC twice daily might be better than once daily. Our data also suggest that higher NTBC concentrations are related to lower SA concentrations. Before stronger conclusions can be drawn, further studies with more data on the dosing of NTBC and related SA concentrations are needed. Therefore, it is mandatory to monitor NTBC and SA concentrations frequently, preferably by home blood spot sampling for which measurement can be performed in expert centers for this disease.

## Electronic supplementary material

ESM 1 (DOCX 18 kb)
