# Supplementary material for: Daily variation of NTBC and its relation to succinylacetone in tyrosinemia type 1 patients comparing a single dose to two doses a day
Source: J Inherit Metab Dis. 2017 Nov 23;41(2):181–6. doi: 10.1007/s10545-017-0112-9 (PMC5830494; doi:10.1007/s10545-017-0112-9)
Supplement: Supplementary file 1 — (DOCX 18 kb) [file 10545_2017_112_MOESM1_ESM.docx]

**Supplementary Material**

**Detailed information of the method used for the determination of SA and NTBC**

Materials
Materials for analyses for Succinylacetone were obtained as described by Johnson et al (2007) and for dimethylsulfoxide, NTBC, mesotrione (internal standard), Girard’s reagent T and acetonitril as described by Herebian et al (2009). Calibrators were prepared in Li-heparin anti-coagulated blood and spotted on filter paper (TFN Grade, Sartorius Stedim DEU). All blood spots were stored in a sealed plastic bag with a silica sachet at ‑20°C until analysis and analyzed at the University Medical Center Groningen.

Sample preparation
 For NTBC and SA, three mm blood spots of samples, controls and calibrators were punched in a 96-well plate. For NTBC analyses, the punches were mixed with 150 µl internal standard solution (3mM Mesotrione in methanol). After 30 min vortexing, the plate was centrifuged at 5000 rcf.

For SA, 2.5 µl internal standard solution (12.3 µM ^13^C_4_-SA in MilliQ water) and 15 µl Girard-T reagent solution (10 mM in MilliQ:acetonitrile:formic acid (100:100:0.2)) were added. After an overnight incubation at room temperature, 150 µl 0.1% formic acid was added, vortexed for 1 minute, and centrifuged at 5000 rcf. Supernatants for SA and NTBC analyses were transferred to auto sampler vials. 1 ul was injected into the U(H)PLC-MS/MS.

Analyses
Blood spot NTBC and SA concentrations were measured using an ultra-performance liquid chromatography (LC30, Shimadzu, Japan) coupled to a triple quadrupole mass spectrometer (API 4500 QTRAP, Sciex, Canada) with an electrospray ionization source (Sciex). Liquid chromatography tandem mass spectrometry (LC-MS/MS) analyses for NTBC and SA were respectively carried out with a Phenomenex Luna NH2 column (30 x 2mm, 5µm) and a Phenomenex Kinetex Biphenyl column (150 x 4.6 mm, 2.6 µm) (Phenomenex Torrance, USA).

The mobile phase for NTBC consisted of 0.1% v/v formic acid in MilliQ H2O (A) and 0.1% v/v formic acid in acetonitrile (B). The following gradient was applied: from 0-1 min: 90% B, from 1-3 min: 10% B; from 3-7 min: 90% B at 0.4 ml/min. For multiple reaction monitoring the following transitions were used: m/z 329.9 → 218.1 for NTBC and m/z 340.0 → 228.2 for Mesotrione for quantification, m/z 329.9 → 126.1 for confirmation of NTBC, with a dwell time of 100 ms. The mobile phase for SA consisted of 0.1% v/v formic acid in MilliQ H2O (A) and methanol (B). Isocratic elution was applied 60% A and 40% B at a flow rate of 0.8 ml/min. For multiple reaction monitoring, the following transitions were used: m/z 272 → 185 for SA and m/z 276 → 189 for 13C4-SA for quantification, m/z 272 → 213 and m/z 276 → 217 for confirmation of SA respectively, with a dwell time of 50 ms. Detection was performed by positive ion electrospray ionization in multiple reaction monitoring mode. The electrospray ionization source temperature was kept at 750 °C and nitrogen was used as the nebulizing gas at a flow rate of 60 L/h. The ionspray voltage was set at 5500V. Data were analyzed using Analyst 1.6.2 (Sciex).

Validation.

Concentrations of NTBC and SA were quantified using a calibration curve. Calibrators were prepared in Li-heparin anti-coagulated human blood with a range of 0.50 – 100.00 µM for SA and 2.50 – 100.00 µM for NTBC respectively, and spotted on filter paper. The methods proved linear within these ranges with correlation coefficients of at least 0.997 for NTBC and 0.998 for SA over three calibration curves. Human blood samples were spiked at three different concentrations and spotted on filter cards. In these three pool blood spot samples, intra-assay variation was calculated from 10 replicates analyzed in a single analytical run. The intra-assay variation of blood spot SA were 10.4% (1.5 µM), 7.7% (10.9 µM) and 5.7% (54.5 µM) respectively, and for blood spot NTBC 6.2% (8.5 µM), 6.6% (27.4 µM) and 9.3% (98.2 µM). Inter-assay variation was assessed by analyzing the three pool samples at ten different days over a three months’ period, showing variation coefficients of 9.8% (1.4 µM), 5.5% (11.1 µM) and 8.6% (52.2 µM) for blood spot SA and 6.6% (8.3 µM), 8.6% (26.9 µM) and 8.0% (97.8 µM) for blood spot NTBC respectively. Limits of detection (LOD) and limits of quantification (LOQ) were determined based on the signal-to-noise ratio approach, using ratios of three and ten respectively. The LOD/LOQ were 0.2/0.6 µM for blood spot SA and 0.2/0.4 µM for blood spot NTBC.
